# Almost There: Transmission Routes of Bacterial Symbionts between Trophic Levels

**DOI:** 10.1371/journal.pone.0004767

**Published:** 2009-03-10

**Authors:** Elad Chiel, Einat Zchori-Fein, Moshe Inbar, Yuval Gottlieb, Tetsuya Adachi-Hagimori, Suzanne E. Kelly, Mark K. Asplen, Martha S. Hunter

**Affiliations:** 1 Department of Evolutionary and Environmental Biology, University of Haifa, Haifa, Israel; 2 Department of Entomology, Newe-Ya'ar Research Center, ARO, Ramat-Yishai, Israel; 3 Department of Entomology, University of Arizona, Tucson, Arizona, United States of America; 4 Department of Entomology, the Volcani Center, ARO, Beit-Dagan, Israel; 5 Graduate School of Biosphere Sciences, Hiroshima University, Higashi-Hiroshima, Hiroshima, Japan; 6 Department of Entomology, University of Minnesota, St. Paul, Minnesota, United States of America; University of California, Berkeley, United States of America

## Abstract

Many intracellular microbial symbionts of arthropods are strictly vertically transmitted and manipulate their host's reproduction in ways that enhance their own transmission. Rare horizontal transmission events are nonetheless necessary for symbiont spread to novel host lineages. Horizontal transmission has been mostly inferred from phylogenetic studies but the mechanisms of spread are still largely a mystery. Here, we investigated transmission of two distantly related bacterial symbionts – *Rickettsia* and *Hamiltonella* – from their host, the sweet potato whitefly, *Bemisia tabaci*, to three species of whitefly parasitoids: *Eretmocerus emiratus*, *Eretmocerus eremicus* and *Encarsia pergandiella*. We also examined the potential for vertical transmission of these whitefly symbionts between parasitoid generations. Using florescence in situ hybridization (FISH) and transmission electron microscopy we found that *Rickettsia* invades *Eretmocerus* larvae during development in a *Rickettsia*-infected host, persists in adults and in females, reaches the ovaries. However, *Rickettsia* does not appear to penetrate the oocytes, but instead is localized in the follicular epithelial cells only. Consequently, *Rickettsia* is not vertically transmitted in *Eretmocerus* wasps, a result supported by diagnostic polymerase chain reaction (PCR). In contrast, *Rickettsia* proved to be merely transient in the digestive tract of *Encarsia* and was excreted with the meconia before wasp pupation. Adults of all three parasitoid species frequently acquired *Rickettsia* via contact with infected whiteflies, most likely by feeding on the host hemolymph (host feeding), but the rate of infection declined sharply within a few days of wasps being removed from infected whiteflies. In contrast with *Rickettsia*, *Hamiltonella* did not establish in any of the parasitoids tested, and none of the parasitoids acquired *Hamiltonella* by host feeding. This study demonstrates potential routes and barriers to horizontal transmission of symbionts across trophic levels. The possible mechanisms that lead to the differences in transmission of species of symbionts among species of hosts are discussed.

## Introduction

The occurrence of arthropods serving as hosts for bacterial symbionts is very common. Primary, obligate symbionts that provide essential nutrients lacking in the host's diet, are strictly maternally transmitted and show congruent phylogenies with those of their host group [1], [2]. Facultative, secondary symbionts are also transmitted vertically, and promote their own transmission by contributing to host fitness or by manipulating the host's reproduction [3]–[9]. Phylogenetic trees of secondary symbionts are largely incongruent with those of their hosts. This, and the fact that the same secondary symbionts are sometimes found in distantly related hosts, is attributed to rare horizontal transmission events of the symbionts between species [1], [10], [11].

The routes of horizontal transmission are not very well known, although transmission via common host plants and/or common natural enemies has been hypothesized, and phylogenetic evidence for the latter has been provided [12]–[14]. Rare examples of experimentally demonstrated natural ***intra***-specific horizontal transmission include *Arsenophonus* [15], *Wolbachia* [16] and a virus [17] in parasitoids, as well as transmission between mates of the same aphid species [18]. In contrast, documentation of *inter*-specific transmission is almost non-existent. Huigens et al [16] showed horizontal transmission of *Wolbachia* between conspecifics of *Trichogramma kayaki* when developing within the same host. However, attempts to show ***inter***-specific horizontal transmission of *Wolbachia* by the same mechanism, between *Trichogramma* species, resulted in loss of the symbiont from the recipient species within a few generations [19]. In lieu of more natural examples, some microinjection studies have been successful in establishing some new stable associations [20]–[23], yet others have been unsuccessful in establishing novel symbiont-host associations [24]–[25], suggesting limits to the ability of symbionts to colonize the germ line of some hosts. While elegant work has shown how *Wolbachia* colonizes the germ line of a *Drosophila* host following injection of cured individuals [26], why symbionts fail to become established is not understood.

The intimate interaction between hosts and their endo-parasitoids would seem to provide opportunities for horizontal transmission of symbionts, as parasitoid larvae consume nothing but symbiont-contaminated food throughout their development. Yet, to our knowledge, there is no experimental evidence of permanent acquisition of arthropods' symbionts by their natural enemies, hence the notion that inter-specific horizontal transmission is a rare event.

Here we followed transmission routes of symbionts from their host – the sweet potato whitefly, *Bemisia tabaci* – to parasitoids. *Bemisia tabaci* (Gennadius) (Hemiptera: Aleyrodidae) is a minute insect that feeds on phloem sap of numerous host plants and is a major pest of agricultural crops [27]. *Bemisia tabaci* harbors a primary symbiont, *Portiera aleyrodidarum* that most probably produces amino acids lacking in the phloem diet [28]. This primary symbiont is located only within specialized cells – bacteriocytes – that are aggregated in two clusters called bacteriomes [1]. In addition, *B. tabaci* may harbor a variety of secondary symbionts: *Arsenophonus*, *Cardinium*, *Fritschea*, *Hamiltonella*, *Rickettsia* and *Wolbachia* (reviewed in [1], [2]; [29]), whose function is yet mostly unknown. The *B. tabaci* colony used in our study carried only two of those secondary symbionts: *Hamiltonella* and *Rickettsia*. *Hamiltonella* is located inside the bacteriocytes with the primary symbiont, while the *Rickettsia* in our culture is dispersed throughout the hemocoel [30].

Bacteria of the genus *Rickettsia* (α-Proteobacteria) are best known as vector-borne agents of many vertebrate diseases. The more recent discoveries of *Rickettsia* in many different invertebrates, with diverse effects such as reproductive manipulation, heat tolerance and plant disease, suggest the disease-causing members represent a small portion of a much larger group [31]. The *Rickettsia* in *B. tabaci* is most closely related to the pea aphid *Rickettsia*, is found in all developmental stages of the whitefly, and is maternally transmitted [32]. *Rickettsia* is highly prevalent in *B. tabaci* populations [29], but its benefits to the host, if any, are not clear. As a matter of fact, *Rickettsia* was found to inflict some costs on fitness parameters of *B. tabaci* [33], [34]. *Hamiltonella* (γ-Proteobacteria) was described from the pea aphid, *Acyrthosiphon pisum*, where it occurs in various tissues both extra- and intra- cellularly and benefits its host by conferring resistance against parasitoids [5], [6], [35].

*Bemisia tabaci* is attacked by a wide variety of natural enemies, including parasitoids of the genera *Eretmocerus* and *Encarsia* (Hymenoptera: Aphelinidae) [36]. These two genera belong to two different sub-families: *Eretmocerus*, with 16 species recorded from *B. tabaci* [37], is in the Aphelininae subfamily; *Encarsia*, with 344 described species, of which 175 species attack whiteflies, is in the Coccophaginae subfamily [38]. *Eretmocerus* and *Encarsia* also differ markedly in their mode of development: *Eretmocerus* spp. lay a single egg under the host venter (i.e., between the host and leaf) and the first instar penetrates and develops within a vital cellular capsule inside the host [39]. *Encarsia* spp., in contrast, lay the egg directly into the body of their whitefly host [40].

In preliminary screening we found that two species of *Eretmocerus*, *Er. eremicus* (Rose & Zolnerowich) and *Er.* sp. nr. *emiratus* (Zolnerowich & Rose), were both highly infected with a *Rickettsia* that had the same 16S rDNA and citrate synthase gene sequences as the *Rickettsia* in their host, *B. tabaci*. Therefore the current study was initiated to address two key questions:

1. What (if any) are the routes of transmission of *Rickettsia* and *Hamiltonella* from *B. tabaci* to the whitefly's parasitoids?
2. Are symbionts that are acquired by the parasitoids then vertically transmitted to parasitoid offspring?

## Materials and Methods

### Insect colonies

#### 1. Whiteflies

Two *B. tabaci* (biotype B) colonies were used for the study: one that carried *Rickettsia* (R^+^) and one that did not (R^−^). *Rickettsia* in these whiteflies was distributed throughout the hemocoel, the ‘scattered’ phenotype [30]. The presence/absence of *Rickettsia* was routinely monitored by diagnostic PCR, as described below. Additionally, the secondary symbiont *Hamiltonella* was established in all individuals of both colonies. Each colony was reared in a separate room at 27±1°C, ca. 60% RH and 16∶8 L∶D. Both colonies have been maintained for over two years on cowpea plants (*Vigna unguiculata* var. California blackeye).

#### 2. Parasitoids

*Eretmocerus* sp. nr. *emiratus*, *Er. eremicus* and *Encarsia pergandiella* were each reared separately on cowpea plants that were infested with R^+^ *B. tabaci* nymphs as hosts, inside transparent ventilated plastic jars. Both sexes of *Eretmocerus* spp. develop as solitary, primary parasitoids, whereas *Encarsia pergandiella* is an autoparasitoid [41]; females are primary parasitoids of whiteflies and males are hyperparasitic, developing on conspecific or heterospecific immatures. Male *En. pergandiella* were thus produced by exposing *Er. eremicus* larvae and pupae to adult female *En. pergandiella*. All parasitoid cultures were kept in a climate-controlled walk-in chamber (27±1°C, ca. 60% RH and 16∶8 L∶D).

#### 3. Establishment of symbiont-free parasitoid colonies

*Eretmocerus emiratus* and *Er. eremicus* were fed on honey containing 50 mg/ml Rifampicin for 48 hrs and were then released on cowpea plants bearing R^−^ *B. tabaci* nymphs for oviposition. This process was repeated for two consecutive generations. The infection status of the progeny was then checked with PCR and both species were found to be free of *Rickettsia* and *Hamiltonella*, therefore they were continuously reared on R^−^ whiteflies under the conditions described above. *Encarsia pergandiella* was not treated the same way because neither *Rickettsia* nor *Hamiltonella* were detected in adult wasps after development in infected whiteflies.

### Methodology

#### 4. PCR analysis

To extract DNA, individual whiteflies or wasps were ground in a 3 µl droplet of proteinase K solution (20 mg/ml, Invitrogen). The droplet was then transferred into a tube containing 50 µl of sterile 10% Chelex beads (Sigma-Aldrich) in PCR water. The tubes were incubated at 37°C for 1 h, then at 96°C for 8 min and then kept at −20°C until analysis. Two microliters of the DNA lysate were used as a template for PCR reactions. The presence of *Rickettsia* was determined using specific primers for amplifying 16S rDNA gene fragments: 528F [5-ACTAATCTAGAGTGTAGTAGGGGATGATGG-3] and 1044R [5-GTTTTCTTATAGTTCCTGGCATTACCC-3]. PCR conditions were: 95°C for 2 min followed by 35 cycles of 92°C, 30 s; 60°C, 30 s; 72°C, 30 s, and final incubation at 72°C for 5 min. Screening for other *B. tabaci* symbionts, including *Hamiltonella*, was done using the primers and conditions described in [29]. Reactions were carried in a 10 µl volume containing 4 pmol of each primer, 0.01 µmol dNTP's, 1× “Thermopol” buffer and 0.4 units of Taq DNA polymerase (New England Biolabs). PCR products were visualized on 1.5% agarose gel using SYBR-Green (Cambrex Bio Science Rockland Inc.). To verify the identity of the PCR products, bands were eluted, DNA was purified (QIAquick gel purification kit, Qiagen) and sent for direct sequencing at the University of Arizona's sequencing facility. The resulting sequences were compared to known sequences using the BLAST algorithm in NCBI. Sequences from whiteflies and parasitoids were compared to one another using the BLAST 2 Sequence in NCBI.

#### 5. Visualization of *Rickettsia* using Fluorescence In Situ Hybridization (FISH) and Transmission Electron Microscopy (TEM)

FISH of *B. tabaci* parasitized nymphs, and adult parasitoids was performed with *Rickettsia*-specific 16S rRNA DNA probes, as described in [32]. Stained samples were whole mounted and viewed under an IX81Olympus FluoView™500 confocal microscope (Tokyo, Japan). Reproducibility and controls were performed as described in the above reference (at least 20 individuals of each species). Samples of *Er. eremicus* females for TEM were prepared as described by [42] (n = 5 females).

### Experiments and Experimental design

#### 6. Acquisition and maintenance of *Rickettsia* and *Hamiltonella* in whitefly parasitoids

Wasps that developed on *Rickettsia*- and *Hamiltonella*-infected whiteflies were censused for infection. Using a fine needle, approx. 100 pupae of each wasp species were removed from leaves and placed in a glass vial with honey. Samples of the pupae were placed in 96% ethanol for diagnostic PCR. Newly emerged wasps were transferred to a new vial with honey and samples were placed in 96% ethanol. Subsequently, wasps were sampled and placed in ethanol on days 3, 6, 9 and 12 post-eclosion. Infection status was then determined by diagnostic PCR in 10–13 wasps of each species, at each time point.

#### 7. Transmission of symbionts from *B. tabaci* to parasitoids

**Figure 1:**
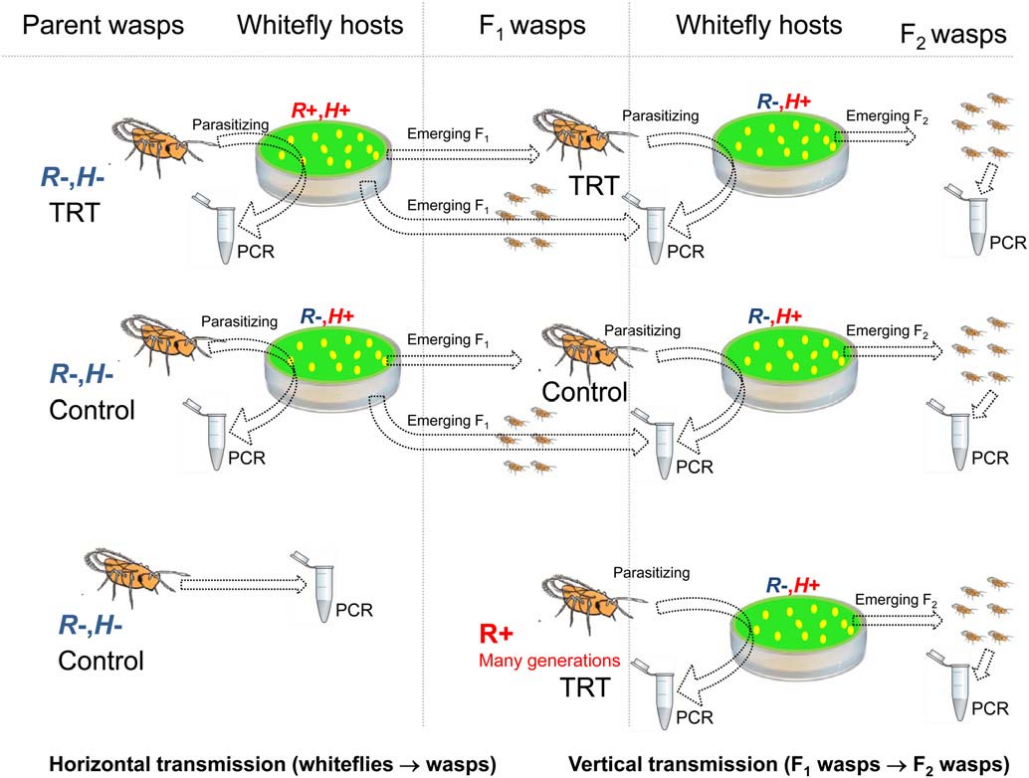
A diagram illustrating the design of experiment 7, transmission of symbionts from *B. tabaci* to parasitoids, and 8, vertical transmission of symbionts in parasitoids. Infection status is indicated either by red “+” sign or blue “−” sign. R = *Rickettsia*, H = *Hamiltonella*. TRT = treatment. Whitefly hosts are illustrated as small yellow ovals on the (green) leaf disks. To test transmission of symbionts from *B. tabaci* to parasitoids, one female parasitoid was introduced to each leaf disk for 24 h, after which they were tested by PCR. From the emerging F_1_, one or two females from each replicate were used to continue to the vertical transmission experiment, while the rest of the cohort was tested by PCR (two-five from each cohort). The emerging F_2_ were all collected and two-five from each cohort were tested by PCR.

There are three likely routes by which symbionts can be transmitted from the whitefly host to its parasitoids: 1) the parasitoid larva acquires symbionts while feeding and developing in an infected host; 2) the adult female wasps acquire symbionts via host-feeding (piercing of the whitefly integument with the ovipositor followed by consumption of host hemolymph); 3) adult wasps might acquire the symbiont via feeding on honeydew secretions of infected whitefly hosts. To test these pathways, cowpea leaf disks (30 mm diameter) infested with 30–50 R^+^ *B. tabaci* nymphs (2^nd^ and 3^rd^ instars) were placed on 1% agar inside 35 mm Petri dishes and sealed with screen lids. One male and one female of R^−^ wasps (cured *Er. emiratus* and *Er. eremicus* grown for six generations on R^−^ whiteflies or R^−^ *En. pergandiella* directly from the culture) were introduced onto each leaf disk for 24 hrs and were then collected to 96% ethanol for PCR analysis. The percentage of infection status of these adults was used to determine the acquisition of the symbiont via either host-feeding or feeding on infected honeydew (scenarios 2 and 3 above). For controls, wasps from the same sources were introduced onto leaf disks bearing R^−^ whiteflies, and some wasps were placed directly in ethanol, without exposure to hosts. The leaf disks bearing parasitized whiteflies were then incubated for approximately two weeks until wasp progeny emergence and then two to five (at least one male and one female) wasps from each disk were collected and placed in 96% ethanol. An estimate of the percentage of symbiont acquisition via exposure during development (scenario 1 above) was determined by the infection status of this second group of wasps. Results were subjected to a chi-square test (JMP 6.1 software, SAS Institute). Figure 1 illustrates the set up of this experiment, as well as the vertical transmission experiment (#8, below).

To study whether symbiont acquisition via host feeding was permanent or transient, another experiment was carried out. Here, approximately 50 R^−^ wasps were introduced onto a plant infested with R^+^ *B. tabaci* nymphs (each species on a separate plant). After 24 h the wasps were retrieved, half of them were transferred directly to 96% ethanol and the other half were kept in glass vials with honey for four days, and then also placed in ethanol. Twenty wasps of each species were screened for *Rickettsia* by PCR: ten wasps from the half that were transferred to ethanol immediately after the exposure to R^+^ whiteflies, and ten from the half that were fed on honey after exposure.

#### 8. Vertical transmission experiments

To study if *Rickettsia* and *Hamiltonella* are vertically transmitted between parasitoid generations, cowpea leaf disks bearing R^−^ *B. tabaci* nymphs were prepared and parasitoids of three treatments were randomly assigned to them: 1) F_1_adults from the previous transmission experiment that were exposed to R^+^ hosts during development, i.e. wasps that have been exposed to the symbionts for one generation only; 2) wasps that had been reared on R^+^ hosts for many generations; 3) adults from the horizontal transmission experiment that emerged from R^−^ hosts (control). One female parasitoid was introduced onto each leaf disk for 24 hrs and was then placed in 96% ethanol for PCR analysis. The leaf disks were incubated for approximately two weeks until progeny emergence and then two to five (at least one male and one female) progeny from each disk were collected and placed in 96% ethanol for PCR analysis. The set up of this experiment is illustrated in Fig. 1.

## Results

### Acquisition and maintenance of *Rickettsia* and *Hamiltonella* in whitefly parasitoids

Almost all pupae of the three studied species carried *Rickettsia* and *Hamiltonella* (*Er. emiratus*- 11 out of 12 infected; *Er. eremicus* – 13/13; *En. pergandiella* females - 10/10; *En. pergandiella* males- 10/10). However, infection of adult wasps differed significantly between the two genera of wasps: *Rickettsia* did not persist in adults of the two *Encarsia* species, while adults of both *Eretmocerus* species were virtually all *Rickettsia*-positive, even 12 days after they had emerged and fed on honey only (sample size = 10 wasps; 9 or 10 tested positive in each sample). In contrast, all *Encarsia* and *Eretmocerus* adults were *Hamiltonella*-negative (0/10 tested for each species).

### Symbiont identity

The sequences obtained from the *Rickettsia* and *Hamiltonella* primers were 99% similar to the sequences of “*Rickettsia* endosymbiont of *Bemisia tabaci*” (DQ077707.1) and “secondary endosymbiont of *Bemisia tabaci* 16S ribosomal RNA gene” (AY429618.1) respectively. The *Rickettsia* 16S rRNA sequences obtained from *B. tabaci* and parasitoids in this study showed 100% similarity.

### Localization of *Rickettsia*

**Figure 2:**
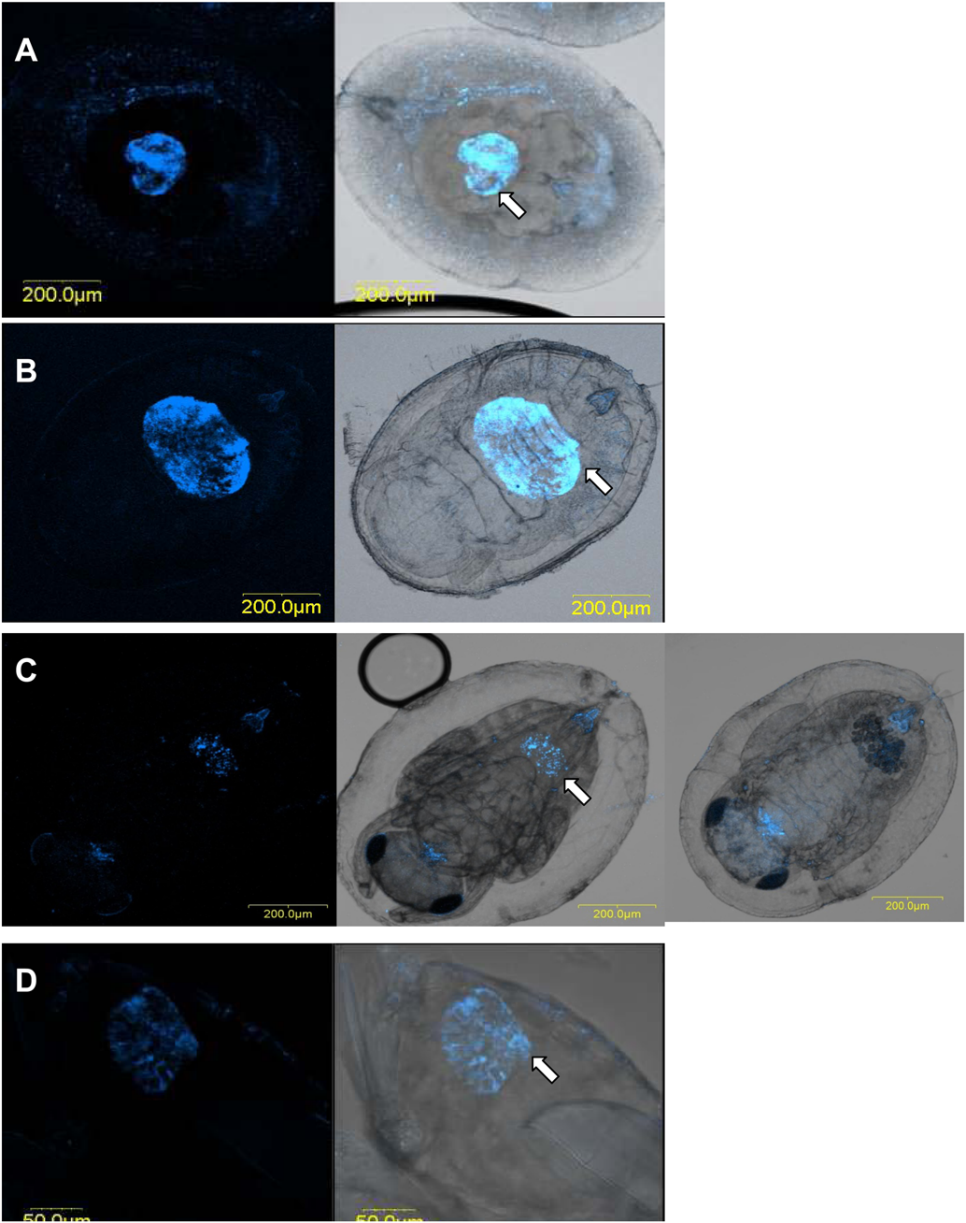
FISH of *Er. emiratus* stained with *Rickettisa* specific probe (blue). Left panel-*Rickettsia* probe fluorescent channel; right panel- overlay of fluorescent and brightfield channels. Arrows pointing to parasitoid gut. A- parasitoid larva (dark, ovoid sphere in the center of the host). Note *Rickettsia* in the parasitoid gut, as well in the whitefly's body remnants, surrounding the parasitoid. B- parasitoid pre-pupa. C- parasitoid pupae (note the autofluorescence of the anus and mouthpart); 1C, right image- brightfield channel only. D- parasitoid adult abdomen.

**Figure 3:**
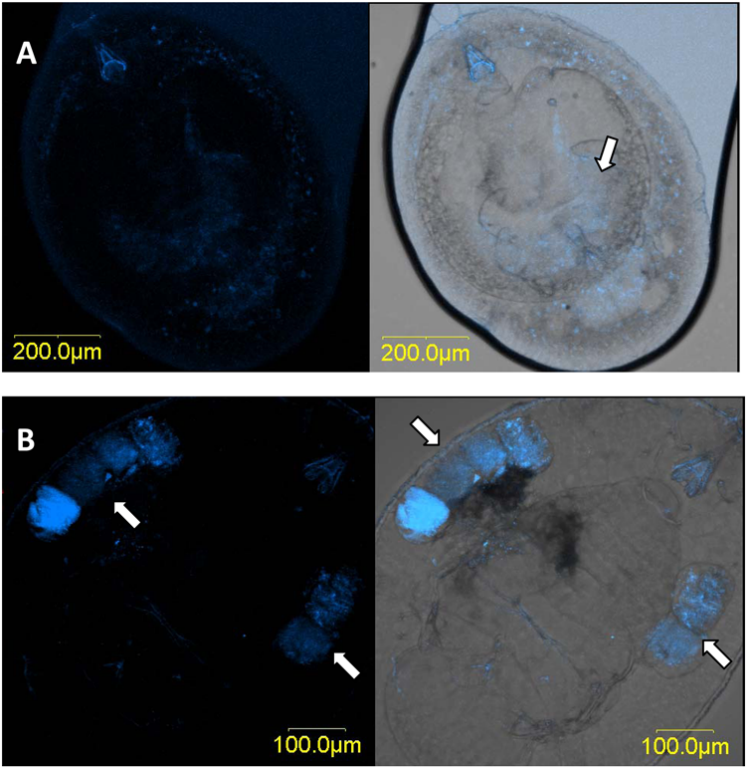
FISH of *En. pergandiella* stained with *Rickettisa* specific probe (blue). Left panel-*Rickettsia* probe fluorescent channel; right panel- overlay of fluorescent and brightfield channels. A- parasitoid larvae, arrow points to specific signal inside the larva body. B- parasitoid pupa, arrows pointing to the meconia deposited outside the parasitoid's body.

Examination of the symbionts' localization by means of FISH shows a concentration of *Rickettsia* in the center of the *Eretmocerus* spp larval body in what seems to be the parasitoid's digestive tract, as well as scattered signals outside of the larval body in the remaining whitefly hemolymph (Fig. 2A). Later on, in the pupal stage, *Rickettsia* is aggregated in a kidney (or oval) shape within the wasp larva, and is more distal, toward the tip of the abdomen (Fig. 2B). Looking at an image without fluorescence shows an identical kidney-shaped concentration of small, dark spheres that are likely meconia (fecal material, typically retained within the wasp body until late in development) (Fig. 2C). In *En. pergandiella*, *Rickettsia* signals can be seen along the digestive tract of the crescent-shaped third instar larva as well as outside of the larva (Fig. 3A). In the pupal stage, however, *Rickettsia* is clearly present only in the meconia, deposited before pupation on both sides of the pre-pupal wasp (Fig. 3B). These FISH results are consistent with the results of the acquisition and sustainability experiment. In particular, they support the finding that adult *En. pergandiella* that developed on R^+^ whiteflies are not infected, and suggest that the detection of *Rickettsia* in pupal *En. pergandiella* by PCR is likely due to an extraction method that includes the whitefly cuticle and meconial pellets that surrounds the pupal wasp.

**Figure 4:**
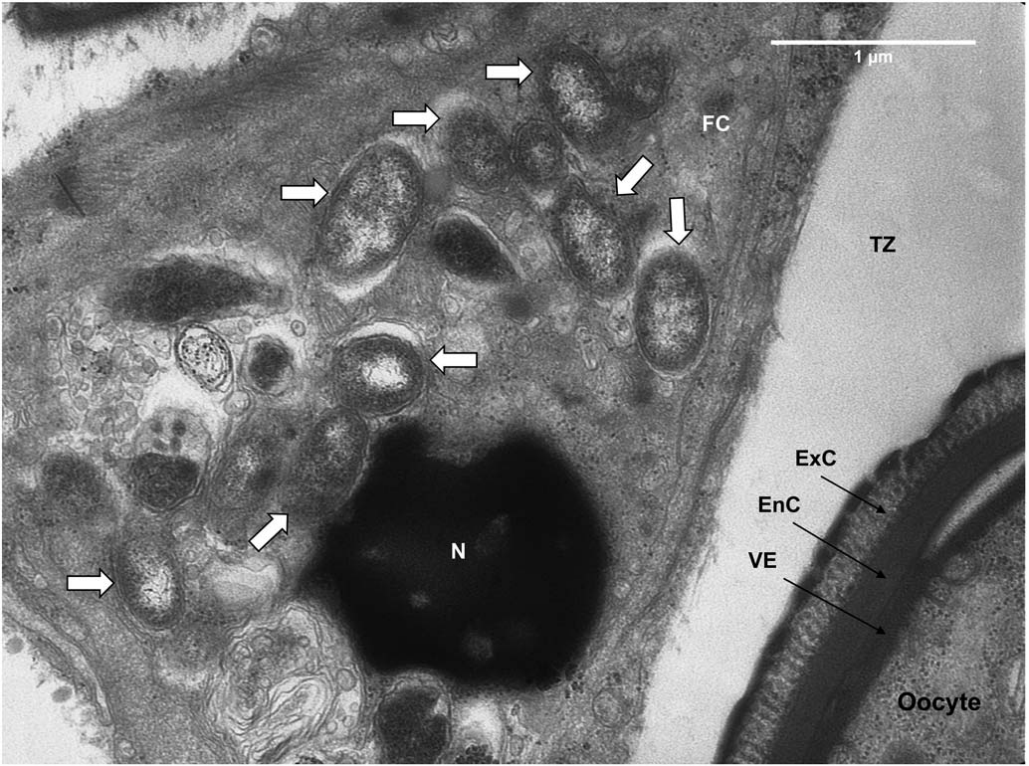
*Rickettsia* (white arrows) in *Er. eremicus* follicular epithelial cell (FC). The gap between the follicular epithelial cell and the oocyte (the transition zone - TZ) is due to oocyte resorption. N-nucleus; EnC- endochorion; ExC- Exochorion; VE- Vitellin envelope.

**Figure 5 pone-0004767-g005:**
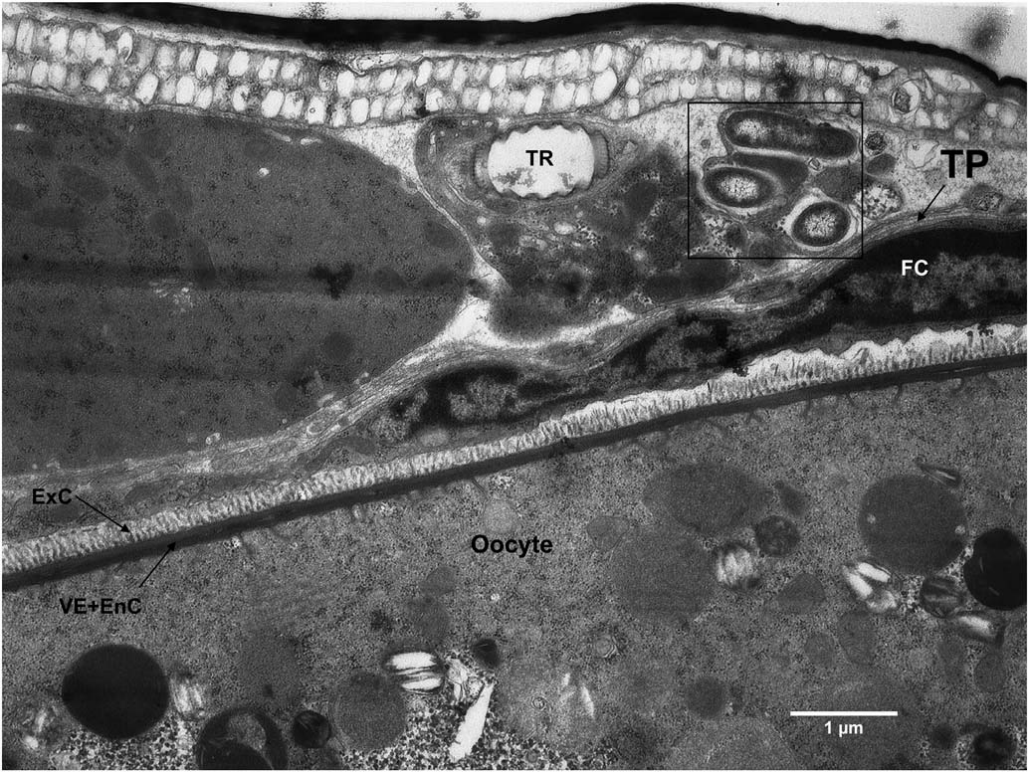
*Rickettsia* (bordered) outside *Er. eremicus* ovary envelope, the tunica propria (TP). FC- follicular epithelial cell; EnC- endochorion; ExC- Exochorion; VE- Vitellin envelope; Tr- Trachea.

**Figure 6:**
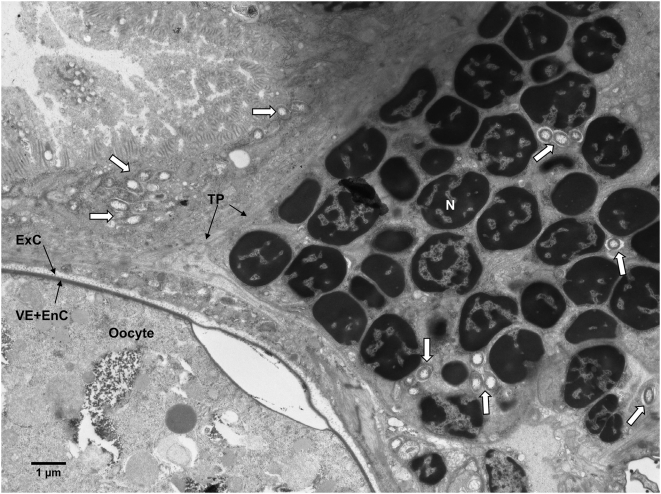
*Rickettsia* (white arrows) in *Er. eremicus* germarium area, between nuclei of stem/pre-follicle/nurse cells as well as outside the ovary, next to the Tunica propria (TP). Note mature oocyte on the bottom left corner area. N-nucleus; EnC- endochorion; ExC- Exochorion; VE- Vitellin envelope.

Electron micrographs of *Er. eremicus* reveal the presence of bacteria inside the ovaries, within follicular epithelial cells, but not within the oocytes (Fig. 4). Bacteria were also seen right outside the ovary, adjacent to the tunica propria, the ovarian envelope (Fig. 5). The germarium also shows bacteria among stem-, pre-follicle-, and nurse cell nuclei (Fig. 6). The determination that these bacteria are *Rickettsia* is supported by: 1) Denaturating gradient gel electrophoresis (DGGE) analysis of the bacteria present in *Er. eremicus* using general 16S rRNA primers that target most known bacteria. A single band, corresponding to *Rickettsia* was found in this analysis (data not shown). 2) Diagnostic PCR using specific primers designed for *B. tabaci* symbionts (*Hamiltonella*, *Wolbachia*, *Cardinium*, *Arsenophonus* and *Rickettsia*) showed bands only for *Rickettsia* in the *Er. eremicus*, as well as for the positive controls in all other cases (data not shown).

### Transmission from *B. tabaci* to parasitoids

#### Eretmocerus

**Figure 7:**
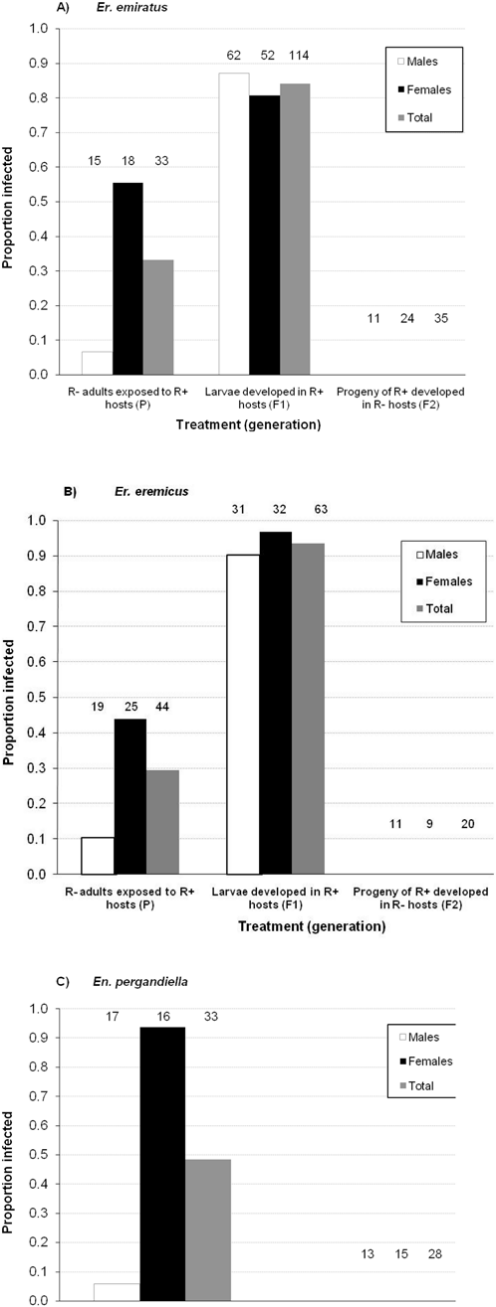
Horizontal transmission (from *R^+^* whiteflies to wasps) and vertical transmission (from *R^+^*wasps to progeny) of *Rickettsia* to males and females of *Er. emiratus* (top), *Er. eremicus* (middle) and *En. pergandiella* (bottom). ‘P’ are *R^−^* wasps that were exposed to *R^+^* whiteflies for 24 hrs (horizontal transmission via host feeding and/or honeydew), ‘F_1_’ are their resulting progeny that developed in *R^+^* hosts (also horizontal transmission), and ‘F_2_’ are progeny of F_1_ that were exposed to *R^−^* hosts (vertical transmission). The numbers above the columns are the sample size, *n*, from which the proportion of infected wasps was calculated. See also Fig. 1 for this experiment's set-up.

Approximately 30% of the uninfected (R^−^) *Eretmocerus* adult wasps (from both species) that were exposed to R^+^ whiteflies as adults were subsequently infected with *Rickettsia* (Fig. 7A & 7B). The proportion of infected females was significantly higher than the proportion of infected males (*Er. emiratus*: 56% infected females vs. 6.7% infected males, χ^2^_32_ = 8.8, P<0.01; *Er. eremicus*: 44% infected females vs. 11% infected males, χ^2^_43_ = 5.8, P = 0.016), suggesting that host-feeding, in which females pierce hosts with their ovipositor and imbibe host hemolymph, is more likely a source of *Rickettsia* than feeding on honeydew (which both sexes do) or simple contact with contaminated insect surfaces. A much higher proportion of those wasps that developed inside R^+^ whiteflies were infected: 84% of *Er. emiratus* and 93% of *Er. eremicus* emerged as *Rickettsia* infected wasps (Fig. 7A & 7B). Thus, transmission of *Rickettsia* from infected whitefly hosts to *Eretmocerus* occurred at the greatest rate during parasitoid development, and to a much lower extent via host feeding by adults. All of the controls, i.e. R^−^ wasps that were not exposed to any hosts and R^−^ wasps that were exposed to R^−^ hosts, were *Rickettsia*-free. *Rickettsia* infections that were acquired by host feeding seemed to be largely transient, as the proportion of infected females decreased sharply four days after removal from hosts (*Er. emiratus*: 15/20 infected immediately after exposure to hosts, vs. 2/10 infected four days later; *Er. eremicus*: 19/20 and 1/10 infected at the two time points, respectively). In contrast with the pattern seen for *Rickettsia*, *Hamiltonella* was not detected in any of the *Eretmocerus* wasps that fed or developed on *Hamiltonella*-infected whiteflies (0/26 tested).

#### En. Pergandiella

Almost all (15 out of 16) of the adult females were infected with *Rickettsia* after exposure to R^+^ hosts, compared to only one infected male (χ^2^_32_ = 25.5, P<0.0001) (Fig. 7C). *Rickettsia* acquired by host feeding and exposure to honeydew was also transient in *En. pergandiella* female adults: 17/20 were infected after exposure to R^+^ hosts, while 0/10 were infected four days later. None of the wasps that developed inside an R^+^ host were infected. As was found in *Eretmocerus*, *Hamiltonella* was also not detected in any of the *En. pergandiella* wasps exposed to infected whiteflies (0/23 tested).

### Vertical transmission

*Eretmocerus* wasps that developed inside R^−^ whitefly hosts emerged as uninfected wasps, even when their mothers were infected throughout their lifetime (0/20 *Er. eremicus*, 0/35 *Er. emiratus* infected, Fig. 7 A, B). There was no difference between the two experimental treatment groups, i.e., wasps with multiple generations of exposure to infected whiteflies prior to the experiment, and wasps with a single generation of exposure (parents). These experiments provide no evidence of vertical transmission of *Rickettsia*. Vertical transmission was not tested in *En. pergandiella* because *Rickettsia* infection did not persist in the adults of this species.

## Discussion

Interspecific horizontal transmission of facultative intracellular symbionts is believed to occur rarely, and little empirical evidence of such transfers exist [e.g. 20], [21], [23]. That horizontal transmission between species must have occurred, however, is amply demonstrated in phylogenetic studies that show little concordance between host and symbiont phylogenies [1], [2]. The results presented here demonstrate distinct transmission patterns of secondary symbionts between trophic levels and reveal differences in those patterns between two closely related parasitoid host genera. Further, we show that *Rickettsia* that is ingested during wasp larval development may penetrate the host hemocoel and infect the ovaries, but do not appear to invade the developing oocytes (Figs. 4–6), preventing vertical transmission in the wasp.

### Horizontal transmission

The variation we document in the transmission of *Rickettsia* from whiteflies to parasitoids highlights two possible views of horizontal transmission. From an evolutionary point of view (most often used in the symbiont literature), our results show no transmission of secondary symbionts from *B. tabaci* to parasitoids that result in a heritable infection. From a mechanistic point of view, however, we document the transmission of a microorganism from one individual to another, unrelated, individual within the same generation, a necessary precondition of a novel heritable infection in a population. Further, we show that symbionts acquired by feeding may be ultimately excreted (“contamination”), or invade the hemocoel and persist throughout the host lifetime, two distinct and sequential steps in the establishment of a long term association.

*Rickettsia* established a transtadial infection in *Eretmocerus* wasps, i.e. *Rickettsia* sustained in *Eretmocerus* from the larval stage through adulthood, but was not transmitted vertically. The FISH results indicate that *Rickettsia* was concentrated in the lower abdomen of the adult *Eretmocerus* wasps (Fig. 2C & 2D). The electron micrographs show that *Rickettsia* reached the ovaries of *Eretmocerus* but did not penetrate the germ line. Instead, it was found in the follicular epithelium surrounding the eggs and also in tissues abutting the ovaries (Figs. 4–6). The fact that *Rickettsia* is found within or in close proximity to the ovaries suggests that like other vertically transmitted bacteria, *Rickettsia* requires admission to the germ line for its spread and persistence in host insect populations. In their thorough study, Frydman et al [26] found that injected *Wolbachia* migrate and enter the *Drosophila* germline via the somatic stem cell niche in the germarium, from which follicular epithelial cells develop. Our results suggest, for *Rickettsia* at least, that invading the oocyte may require an adaptation distinct from the ability to find and invade the ovaries. Nonetheless, the inability of *Rickettsia* to invade the germ line of *Eretmocerus* may be a result of a defense mechanism of the latter.

The frequency of interspecific horizontal transmission in endosymbiosis of arthropods is clearly low and variable (excluding disease agents vectored by ticks etc). Possibly, the paucity of empirical studies conceals a number of unpublished negative results. Among published results, the frequency of *Wolbachia* horizontal transfer between *Trichogramma* species sharing a common host was 0–40% and the vertical transmission within the recipient species diminished within a few generations [19]. Similarly, *Spiroplasma* was horizontally transmitted between two species of *Drosophila* by an ectoparasitic mite vector but the subsequent vertical transmission was very low [43]. Variability of interspecific transmission success was also demonstrated in the study of Russell & Moran [25]: pea aphids were injected with three different symbionts that were obtained from other aphid species. Two symbionts - *Hamiltonella* and *Arsenophonus* - were successfully established and maintained for multiple generations in their new host, whereas the third one – *Regiella* – was not. Grenier et al [22] reported successful horizontal transfer of *Wolbachia* from one species of *Trichogramma* to another via microinjection, followed by stable vertical transmission, but the efficiency of this process was low. To the best of our knowledge, the only study that describes symbiont horizontal transmission from a host to its parasitoid is that of Heath et al. [44], in which *Wolbachia* was weakly transmitted (3.2%) from an infected *Drosophila* host to a parasitoid, and subsequently diminished within four generations. Compared to these studies, the efficiency of *Rickettsia* transmission from the host, *B. tabaci*, to *Eretmocerus* wasps was very high and yet no vertical transmission was observed. Our results therefore support the notion that invasion of the germ line may be the greatest challenge for symbionts invading novel hosts. Among parasitoids, maternally transmitted *Rickettsia* was so far only found in a leaf miner parasitoid, where it causes parthenogenesis. However, it is not known whether this symbiont is present also in its hosts, which may be indicative of inter-trophic horizontal transmission [45].

### Differences among hosts

Why does *Rickettsia* establish (even if for only one generation) in the *Eretmocerus* adults, whereas it appears to be completely excreted by the *Encarsia*? *Encarsia* embryos and larvae are in intimate, direct contact with the host's hemolymph throughout their development, whereas *Eretmocerus* become in contact with the host's hemolymph only in the third instar, due to the unique capsule in which the larval wasps reside [39]. Hence, our finding that *Eretmocerus* acquire *Rickettsia* while *Encarsia* do not is, at first, counterintuitive. It is possible that these differences relate to the timing of the deposition of the meconium, fecal material. In *En. pergandiella* the mid-gut and the hind-gut are not continuous in early development, when the larva is in a fluid environment, but join only at the end of the third instar stage. Subsequently, the prepupal wasp deposits the meconium, with *Rickettsia* in it, and then pupates [40]. *Eretmocerus* spp., in contrast, excrete the meconium only after the adult emerges, so meconia, with *Rickettsia* in them, are present in the body throughout metamorphosis. It may be that *Rickettsia* has the opportunity to invade new tissues during this phase, when tissues are breaking down and new ones are being built. This idea is supported by the observation that adult acquisition of the symbiont by consumption of honeydew or host hemolymph does not persist. Nevertheless, other routes of infection cannot be excluded: *Rickettsia* may get to the ovaries by crossing the larval mid-gut tissues, which in aphelinid larvae typically bear very few cells, no typical epithelium and no membranes (Dan Gerling, pers. comm.).

### Differences between symbionts

Adult wasps of all species in our study acquired *Rickettsia* but not *Hamiltonella* from host feeding. One possible reason for that difference may be the localization of the two symbionts: *Rickettsia* is abundant and accessible in the host hemolymph consumed during the process of host-feeding while *Hamiltonella* is sequestered within the bacteriomes [30]. Another explanation is required, however, for why *Eretmocerus*, during their development inside a host, acquire *Rickettsia* but not *Hamiltonella*, since the parasitoid larvae consume the entire host contents before pupation. Indeed, it seems that *Rickettsia* is generally more prone to horizontal transmission (e.g. to mammalian hosts in the case of the disease agents, or to plants in the case of insect-vectored plant pathogens) than many facultative intracellular symbiont lineages. To date, *Rickettsia* has been found in many host lineages [2], [31], whereas *Hamiltonella* has so far been revealed only in aphids, in whiteflies and in one psyllid species [2], [10]. A possible mechanism for a greater propensity for horizontal transmission is greater symbiont mobility: while little is known about mobility in most symbiont lineages, some *Rickettsia* are able to move between cells and tissues using actin filaments [46], [47]. We know nothing about the mobility of *Hamiltonella*, yet the fact that *Hamiltonella* reside in *B. tabaci* bacteriocytes where they are vertically transmitted along with the primary symbiont *Portiera*, suggests that *Hamiltonella* may have more limited mobility.

Naturally, an interesting question for further research would be to look for phenotypic effects of *Rickettsia* infection on the parasitoids. Preliminary results showed no differences between R+ and R− *Er. emiratus* with regards to fecundity, longevity and sex ratio, thus more fitness parameters need to be explored to address this question (Chiel et al., unpublished results).

To conclude, our study is one of few empirical demonstrations of the routes and barriers to horizontal transmission of facultative symbionts. These data are especially relevant to the often repeated idea that parasitoids or predators may be instrumental agents for moving symbionts from one host lineage to the next. In fact, this notion has some phylogenetic support [e.g. 12], [48], but in some cases, enemies have likely been wrongly diagnosed by PCR as being stably infected when the symbionts are simply present in the gut along with the prey or host material [49]. Our data suggest that host-parasitoid transmission may, nonetheless, be one way in which symbionts acquire new hosts. Given that the symbiont is on the doorstep of vertical transmission, it is not hard to imagine that some lineage might, with time, acquire an adaptation that improves the precision of cell targeting in this new host lineage to get the symbiont over the threshold. Lastly, our study underscores how little we currently know about the processes of dispersal of symbionts to new host lineages, and the within-host movement and germ-line invasion processes necessary for them to stay once they get there.
